# Effect of Oral Sodium Bicarbonate Treatment on 24-Hour Ambulatory Blood Pressure Measurements in Patients With Chronic Kidney Disease and Metabolic Acidosis

**DOI:** 10.3389/fmed.2021.711034

**Published:** 2021-09-06

**Authors:** Martina Gaggl, Alexandra Repitz, Sonja Riesenhuber, Christof Aigner, Christopher Sliber, Melanie Fraunschiel, Daniel Cejka, Gere Sunder-Plassmann

**Affiliations:** ^1^Division of Nephrology and Dialysis, Department of Medicine III, Medical University of Vienna, Vienna, Austria; ^2^Department of Medicine, Sana Klinikum Offenbach, Offenbach, Germany; ^3^ITSC – IT Systems & Communications, Section IT4Science, Medical University of Vienna, Vienna, Austria; ^4^Department of Medicine III, Nephrology, Hypertension, Transplantation and Rheumatology, Ordensklinikum Linz – Elisabethinen, Linz, Austria

**Keywords:** metabolic acidosis, sodium bicarbonate, blood pressure, 24-hour ambulatory blood pressure measurement, chronic kidney disease

## Abstract

**Background:** Sodium bicarbonate supplementation is a mainstay in the treatment of metabolic acidosis in patients with chronic kidney disease (CKD). Recent studies showed reduction of progression of CKD and reduced all-cause mortality. However, additional sodium loading could worsen arterial hypertension, a well-known contributor to progression of CKD. This patient-relevant and economically negative side effect is under-studied in prospective studies up until now.

**Objective:** The aim of this study was to analyze the effect of sodium bicarbonate treatment on arterial blood pressure at baseline and after 8 weeks.

**Methods:** The SoBic study is an ongoing randomized controlled trial, in which patients with CKD receive either a high dose of oral sodium bicarbonate or a rescue treatment, if necessary. We used standardized office blood pressure and 24-hour ambulatory blood pressure monitoring (24h-ABPM). Regression models were adjusted for estimated glomerular filtration rate and change of antihypertensives.

**Results:** 47 subjects were enrolled and the mean age was 57 (±14.6) years and 18 (38%) were female. In 43 randomized subjects with sufficiently performed 24h-ABPM neither systolic 24h-ABPM (2.522; 95%CI: −2.364, 7.408; mmHg) nor diastolic 24h-ABPM (0.868; 95%CI: −2.411, 4.147; mmHg) was affected by study group allocation. When looking at the effect of individual sodium bicarbonate dose on 24h-ABPM, the fully adjusted model suggested an increase of 0.047 (95%CI: −0.026, 0.119) mmHg by each mg/kg *per* day increase of sodium bicarbonate dose.

**Conclusion:** Sodium bicarbonate supplementation over 8 weeks did not significantly increase blood pressure measured by 24h-ABPM in CKD patients.

**Trial Registration:** EUDRACT Number: 2012-001824-36; 12/07/2012 (https://www.clinicaltrialsregister.eu).

## Introduction

Metabolic acidosis regularly accompanies chronic kidney disease (CKD) and on long-term enhances several detrimental pathways leading to nephron loss and renal fibrosis. Evidence now exists showing that supplementing alkali can halt progression of CKD and more importantly can reduce all-cause mortality (1–3). A recent meta-analysis underscored these benefits, but also reported worsening of arterial hypertension or the requirement of increased antihypertensive therapy (4), another well-established contributor to progression of CKD. Sodium bicarbonate, the most frequently used alkaline supplement, results in substantial sodium loading in addition to alimentary sodium intake. Recently Bushinsky (5) discussed the adverse effect of sodium loading accompanied by alkali supplementation on arterial blood pressure and raised caution. This patient-relevant and economically negative side effect is under-studied in prospective studies up until now.

The SoBic study is an ongoing randomized controlled trial, in which patients with CKD receive either a high dose of oral sodium bicarbonate or a rescue dose, if necessary. The study protocol includes standardized office blood pressure (BP) measurements and 24-hour ambulatory blood pressure monitoring (24h-ABPM), the gold standard of BP monitoring. The aim of the study was to analyze the effect of sodium bicarbonate loading on arterial BP assessed by 24h-ABPM at baseline and after 8 weeks of sodium bicarbonate treatment. We hypothesized that sodium bicarbonate treatment has a dose-dependent effect on change in arterial BP from baseline to week 8.

## Materials and Methods

### Study Design and Participants

The SoBic study is a single-center, randomized controlled trial performed at the Nephrological outpatient service of the Medical University of Vienna. The study is described in detail elsewhere (6). In brief, patients with CKD stage G3 and G4 and chronic metabolic acidosis (serum ${\text{HCO}}_{3}^{-}$ ≤21 mmol/L) were randomized to either receive a high dose of oral sodium bicarbonate with a serum target ${\text{HCO}}_{3}^{-}$ level of 24 ± 1 mmol/L (intervention group) or receive a rescue therapy of sodium bicarbonate, if necessary, with a serum target level of 20 ± 1 mmol/L (rescue group). Study personnel and subjects were aware of the randomization assignment. Inclusion criteria were age over 18 years, renal function (measured by estimated glomerular filtration rate (eGFR) calculated by the four-variable Modification of Diet in Renal Disease (MDRD) study equation) between 60 and 15 mL/min *per* 1.73 m^2^, a venous serum ${\text{HCO}}_{3}^{-}$ of ≤21 mmol/L on two consecutive measurements (at least 1 day apart), and stable clinical condition of the patient. Exclusion criteria were malignant disease or <5 years of successful treatment of malignant disease (except dermal malignancies and carcinoma *in situ* of the cervix declared to be cured), patients with morbid obesity (body mass index >40 kg/m^2^), chronic inflammation (C reactive protein >10 mg/dL), immunosuppressive therapy of any kind, poorly controlled blood pressure (>150/90 mmHg despite the use of four agents), overt congestive heart failure, or known peanut or soy allergy (as given by the manufacturer of the study medication).

All methods were carried out in accordance with relevant guidelines and regulations.

The ethics committee of the Medical University of Vienna approved the study (EK Number: 1331/2012), the trial was registered at the EU registry for clinical studies (EUDRACT Number: 2012-001824-36; 12/07/2012; https://www.clinicaltrialsregister.eu), and all subjects gave written informed consent.

For the present study we included all subjects randomized until March 2019. Patients had office BP measurements at each visit and a 24h-ABPM at baseline and after 8 weeks. We hypothesized that sodium bicarbonate treatment has a dose-dependent effect on systolic (SBP) and/or diastolic BP (DBP). First, we compared change of mean 24h-ABPM (overall, day-time, and night-time) from baseline to week 8 between the intervention and the rescue group. Because the design of the SoBic study allows rescue sodium bicarbonate treatment in controls if necessary, we second investigated the change in 24h-ABPM from baseline to week 8 with respect to individual sodium bicarbonate dose (mg) per kilogram (kg) body weight *per* day. Third, we estimated the change in 24h-ABPM from baseline to week 8 with respect to low dose, moderate dose, and high sodium bicarbonate dose vs. no treatment independent of randomization.

The primary outcome was change in arterial BP (SBP and DBP) at week 8 compared to baseline. We defined that a mean change of equal or >7 mmHg in SBP (overall systolic 24h-ABPM) between baseline and week 8 is patient-relevant, as this represents the average SBP-lowering effect of approximately one antihypertensive agent (7). With a defined power of 80% and a type-1 error of 5% we would have to enroll 18 patients or more per group to be able to observe a change of 7 mmHg, if the null hypothesis was true.

### Laboratory Measurements and Clinical Assessment

Serum creatinine was determined by means of the Jaffe method (IDMS-traceable, reference range <1.2 mg/dL, mg/dL × 88.4 = μmol/L). Bicarbonate measurements were performed in venous blood samples and determined by an ABL 700 Copenhagen Blood Gas Analyzer (Radiometer Copenhagen, Copenhagen, Denmark). Serum and urine electrolytes were assessed as detailed elsewhere (6). Urine pH has been measured by dipstick (5, 5.5, 6, 6.5, 7) analyzed by a validated device.

Body weight was measured on a validated scale each visit and presence of edema (yes/no) or worsening of present edema (yes/no) was assessed by an experienced clinician. Change in concomitant medication, especially antihypertensives and diuretics, was monitored at each visit. Number of antihypertensives is given on average (total number of antihypertensives/N) overall and per type of antihypertensive. Initiation of an additional antihypertensive/diuretic or increase of dosage is presented as “increase in antihypertensives/diuretics” (yes/no). Decrease of dose or cessation of an antihypertensive is presented as “decrease in antihypertensives/diuretics” (yes/no).

### Sodium Bicarbonate

Study medication was supplied as sodium bicarbonate capsules à 840 mg in an extended-release product (Nephrotrans 840 mg, Medice Arzneimittel Pütter GmbH & Co. Iserlohn, Germany). One capsule contained 840 mg sodium bicarbonate (NaHCO_3_; 10.08 meq) and includes 230.16 mg of sodium. At each visit dosage could be modified according to the treatment algorithm and allocated study group, as detailed in Gaggl et al. (6) Compliance was measured by pill count each visit. The presented dose is the reported medication taken during the last 7 days prior to the visit at week 8, ideally as prescribed at visit 4 by the investigator. Sodium bicarbonate dose was presented as mg *per* kg body weight (at baseline) *per* day and as mg *per* day (effective dose). For further analysis we categorized subjects into treatment categories: no treatment, low dose (1–37.9 mg/kg/day), moderate dose (38–79.9 mg/kg/day), and high dose (≥80 mg/kg/day) of sodium bicarbonate. Boundaries for treatment categories were chosen to reflect the size of the effects expected across the range of exposure (8).

### Blood Pressure Measurements

#### Office Blood Pressure Measurements

At each visit office BP was taken by a trained study nurse using a Boso medicus uno (Bosch + Sohn GmbH u. Co. KG, Jungingen, Germany) device. Measurements were taken 3 times at least 1 min apart after a rest of at least 10 min in a sitting position. The mean SBP and DBP value, respectively, of the 3 measurements was reported (mmHg). Measurements are summarized as mean SBP (mSBP) and DBP (mDBP) overall and for respective study groups. Differences in SBP and DBP were calculated by subtracting the baseline from week 8 measurement (mean Δ office BP = mean office BP_week8_ – mean office BP_baseline_).

#### 24-Hour Ambulatory Blood Pressure Measurements

Twenty Four hours-ABPM was measured by oscillometry using a Mobil-O-Graph NG (I.E.A. GmbH, Stolberg, Germany) device at 15-min interval during the daytime (between 8:00–21:59) and 30-min interval during nighttime (between 22:00–7:59). Measurements were performed according to the guidelines of the European Society of Hypertension (9). We defined 24h-ABPM valid if more than 23 measurements during daytime and 11 during nighttime (random draw) were successful. A recent validation study showed that those are the minimum number of measurements needed to achieve a precision of ±2.5 mmHg in blood pressure monitoring (10). Total number of measurements was summarized and the overall (24h-ABPM), daytime (daytime-ABPM) and nighttime mean (nighttime-ABPM) and standard deviation (±SD) were calculated. Measurements are summarized as mean systolic and diastolic 24h-ABPM overall and for respective study groups. Differences in means were calculated by subtracting the baseline from week 8 measurement (mean **Δ** 24h-ABPM = mean 24h-ABPM _week8_ – mean 24h-ABPM _baseline_).

### Statistical Analysis

Descriptive statistics are given in count (percent), mean (± standard deviation, SD), or median (interquartile range, IQR). Descriptive data is depicted by means of dot plots. Student's *t*-test for paired and unpaired groups were performed, as well as analysis of variances (ANOVA) to compare means across groups. To compare difference in mean Δ office BP and mean Δ 24h-ABPM we calculated unpaired *t*-tests. For not normally distributed data non-parametric tests were used to compare medians.

To assess change in blood pressure, first, we performed linear regression with respect to study group randomization (reference: rescue group; crude) adjusted for change of eGFR (mL/min *per* 1.73 m^2^; adjusted I), and increase of antihypertensive medication (reference: no change), and decrease of antihypertensive medication (reference: no change; adjusted II).

Second, we regressed individual sodium bicarbonate dose (mg/kg *per* day) on change of BP, adjusted as described above.

Third, dose dependent effects on change in BP were estimated by using treatment categories low, moderate, and high dose (reference: no treatment) in crude and adjusted models as given above.

All results are presented as point estimates and corresponding 95% confidence intervals (95%CI), and all results with a *p*-value lower than 0.05 were considered statistically significant. All statistical analysis was performed with R (R Core Team, 2016. “R: A language and environment for statistical computing”; R Foundation for Statistical Computing, Vienna, Austria; https://www.R-project.org/).

## Results

### Participants and Sodium Bicarbonate Dose

**Figure 1:**
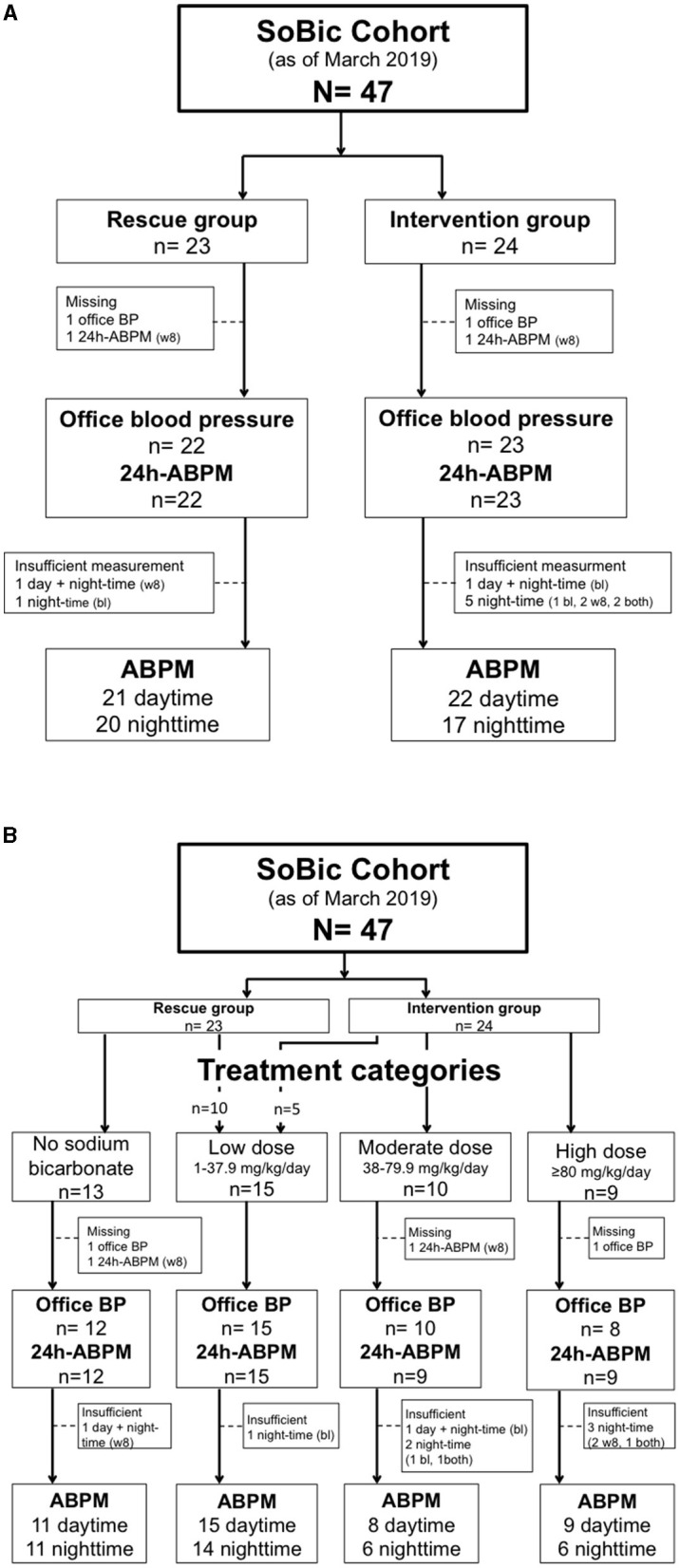
Patient flow **(A)** according to study group and **(B)** according to treatment categories. 24h-ABPM, 24-h blood pressure monitoring; bl, baseline measurement; w8, week 8 measurement.

Until March 2019 47 subjects were enrolled in the SoBic study and had completed week 8 of the study protocol (Figure 1A). The mean age was 57 (±14.6) years and 18 (38%) were females. Ninety-six percent had a diagnosis of arterial hypertension at baseline [median time: 5 (IQR: 3–15) years] and 34% had diabetes [median time: 5.5 (IQR: 2.25–11) years]. Population demographics at baseline are given in Table 1A. No significant differences were observed at baseline across groups. Twenty-four patients were randomized to the intervention group and received a median daily dose of 63 mg/kg *per* day (IQR: 50–88) or an effective median dose of 5,040 mg/day (IQR: 4,200–6,930) at week 8. Of the 23 patients of the rescue group 10 (44%) received sodium bicarbonate (median sodium bicarbonate dose 21 mg/kg *per* day, IQR: 19–31; effective median dose 1,680 mg *per* day, IQR: 1,680–2,520) at week 8. Overall, 13 patients did not take sodium bicarbonate, 15 took a low dose (median: 24.71 mg/kg *per* day, IQR: 20.12–31.17/2,520 mg/day, IQR: 1,680–2,296), 10 a moderate dose (median: 59.94 mg/kg *per* day, IQR: 57.72–63/5,040 mg/day, IQR: 4,200–5,040), and 9 a high dose (median: 89.09 mg/kg *per* day, IQR: 87.91–99.47/6,720 mg/day, IQR: 5,880–7,560) at week 8 (Table 1B).

**Table 1A:** Basic demographic data at baseline [count (percent) and mean (standard deviation), respectively] with respect to study groups.

	**Rescue group**	**Intervention group**
*N*	23 (49%)	24 (51%)
Sex (female)	9 (39%)	9 (38%)
Age (years)	56 (±16.5)	58 (±12.8)
BMI (kg/m^2^)	27.4 (±4.26)	28.8 (±6.22)
eGFR (mL/min *per* 1.73 m^2^)	27 (±9.7)	25.33 (±7.5)
Serum ${\text{HCO}}_{3}^{-}$ (mmol/L)	19.2 (±1.5)	18.8 (±1.8)
Urine sodium (mmol/24 h)	186 (±88)	190 (±79)
Urine pH	5.5 (±1.1)	5.3 (±0.54)
Number of antihypertensives	64 (2.8 per subject)	48 (2.0 per subject)
**Type of antihypertensive**		
Diuretics	15 (65%)	8 (33%)
Calcium antagonist	11 (48%)	10 (42%)
ACEi/ARB	19 (83%)	18 (75%)
Beta blocker	10 (45%)	8 (33%)
Others	9 (39%)	4 (17%)
**Underlying renal disease**		
Glomerulopathy	7 (30%)	5 (21%)
Vascular nephropathy	7 (30%)	4 (17%)
Diabetic nephropathy	6 (26%)	6 (25%)
Others	3 (13%)	9 (38%)
**Comorbidities**		
Arterial hypertension	22 (96%)	23 (96%)
Diabetes	7 (30%)	9 (38 %)
Cardiovascular disease	3 (13%)	4 (17%)
Congestive heart failure	3 (13%)	1 (4%)
Peripheral occlusive disease	4 (17%)	3 (13%)
COPD/Asthma	4 (17%)	0

*BMI, body mass index; eGFR, estimated glomerular filtration rate; ACEi, angiotensin converting enzyme inhibitor; ARB, angiotensin II receptor blocker; COPD, chronic obstructive pulmonary disease*.

**Table 1B:** Basic demographic data at baseline [count (percent), mean (standard deviation)] with respect to treatment category.

	**Treatment category sodium bicarbonate (mg/kg** ***per*** **day)**				
	**None**	**1–37.9**	**38–79**	**≥80**	***p*-value**
*N*	13	15	10	9	
**Study group**					
Intervention	0	5	10	9	
Rescue	13	10	0	0	
Sex (female)	5 (39%)	6 (40%)	1 (10%)	6 (67%)	0.092
Age (years)	56 (±17.3)	58 (±15)	61 (±8.8)	53 (±16)	0.741
BMI (kg/m^2^)	26 (±4.5)	31 (±5.4)	27 (±7)	27 (±2.8)	0.083
eGFR (mL/min *per* 1.73 m^2^)	30.3 (±10.3)	24 (±7.2)	25 (±9.1)	25 (±6.7)	0.338
Serum ${\text{HCO}}_{3}^{-}$ (mmol/L)	20.2 (±0.9)	18.6 (±1.4)	19 (±1.1)	17.9 (±2.3)	0.007
Urine sodium (mmol/24 h)	207 (±95)	167 (±79)	201 (±82)	181 (±75)	0.766
Urine pH	5.7 (1.34)	5.2 (±0.5)	5.2 (±0.3)	5.6 (±0.7)	0.281
Number of antihypertensives	35 (2.7 per subject)	41 (2.7 per subject)	21 (2.1 per subject)	15 (1.7 per subject)	0.001
**Type of antihypertensive**					
Diuretics	8 (62%)	9 (60%)	4 (40%)	2 (22%)	
Calcium antagonist	6 (46%)	8 (53%)	4 (40%)	3 (33%)	
ACEi/ARB	11 (85%)	12 (80%)	7 (70%)	7 (78%)	
Beta blocker	5 (39%)	8 (53%)	3 (30%)	2 (22%)	
Others	5 (39%)	4 (27%)	3 (30%)	1 (11%)	0.994
**Underlying renal disease**					
Glomerulopathy	4 (31%)	3 (20%)	1 (10%)	4 (44%)	
Vascular nephropathy	5 (39%)	2 (13%)	3 (30%)	1 (11%)	
Diabetic nephropathy	3 (23%)	5 (33%)	4 (40%)	0	
Others	1 (8%)	5 (33%)	2 (20%)	4 (44%)	0.200
**Comorbidities**					
Arterial hypertension	12 (92%)	15 (100%)	10 (100%)	8 (89%)	0.436
Diabetes	4 (31%)	4 (27%)	8 (80%)	0	0.002
Cardiovascular disease	2 (15%)	2 (13%)	3 (30%)	0	0.389
Congestive heart failure	2 (15%)	1 (7%)	1 (10%)	0	0.756
Peripheral occlusive disease	2 (15%)	2 (13%)	2 (20%)	1 (11%)	1.000
COPD/Asthma	2 (15%)	2 (13%)	0	0	0.529

*Statistical testing was done by means of the Kruskal-Wallis rank sum test for continuous data and by the Fisher Exact test for categorical data*.

*BMI, body mass index; eGFR, estimated glomerular filtration rate; ACEi, angiotensin converting enzyme inhibitor; ARB, angiotensin II receptor blocker; COPD, chronic obstructive pulmonary disease*.

### Effect of Sodium Bicarbonate on Blood Pressure

Mean office SBP and DBP at baseline were 129 (±16) and 80 (±13) mmHg and was higher in the intervention group at baseline (**Δ** SBP: 10 mmHg, **Δ** DBP: 8 mmHg; Table 2). Similar results were observed using 24h-ABPM, but the differences were less distinct (**Δ** systolic 24h-ABPM: 3 mmHg, **Δ** diastolic 24h-ABPM: 4 mmHg).

**Table 2A:** Mean office and 24h-ambulatory blood pressure measurements (±SD, mmHg) with respect to study group and point in time, and mean blood pressure change (95%CI, mmHg).

	**Rescue group**				**Intervention group**				**Differences between groups**
	***n***	**Baseline**	**Week 8**	**Change (95% CI)**	**n**	**Baseline**	**Week 8**	**Change (95% CI)**	***p*-value**
**24h-ABPM**	21				22				
Systolic		122 ± 11.7	124 ± 11.6	2.1 (−0.79 to 4.90)		125 ± 9.5	129 ± 11.7	4.58 (0.30 to 8.86)	0.314
Diastolic		76 ± 6.8	77 ± 6.8	1.11 (−1.18 to 3.4)		80 ± 6.2	82 ± 5.8	2.18 (−0.12 to 4.48)	0.606
**Daytime-ABPM**	21				22				
Systolic		123 ± 12.4	125 ± 10.2	2.1 (−1.12 to 5.24)		126 ± 10.0	130 ± 11.4	3.88 (−0.62 to 8.37)	0.500
Diastolic		77 ± 7.0	78 ± 6.8	1.5 (−1.0 to 3.95)		81 ± 6.5	83 ± 6.3	2.46 (−1.9 to 5.10)	0.577
**Nighttime-ABPM**	19				17				
Systolic		119 ± 12.2	120 ± 14.5	1.49 (−1.65 to 4.63)		119 ± 10.7	125 ± 14.7	5.72 (0.75 to 10.70)	0.140
Diastolic		73 ± 8.7	74 ± 9.1	0.70 (−1.75 to 3.15)		75 ± 7.8	77 ± 7.3	2.17 (−0.78 to 5.12)	0.425
**Office BP**	22				23				
Systolic		124 ± 17.0	132 ± 16.1	8.64 (1.00 to 16.28)		134 ± 12.6	135 ± 16.1	1.35 (−4.31 to 7.01)	0.119
Diastolic		76 ± 14.8	80 ± 12.0	6.36 (2.68 to 10.05)		84 ± 10.1	81 ± 15.0	−1.83 (−5.88 to 2.23)	0.003

*Missings: 24h- and daytime-ABPM 1 rescue, 1 intervention group; Nighttime-ABPM: 1 rescue, 5 intervention group; Office BP; 1 rescue, 1 intervention group*.

*Change between baseline and week 8 was tested by a paired t-test, difference in change across groups was tested by an unpaired t-test*.

*24h-ABPM, 24 h ambulatory blood pressure monitoring; Daytime ABPM, from 8:00 until 22:00 ambulatory blood pressure monitoring; Nighttime ABPM, from 22:01 until 7:59 ambulatory blood pressure monitoring; Office BP, office blood pressure measurement; 95% CI, 95 % confidence interval*.

At baseline on average 2.4 antihypertensives were prescribed per subject, which was higher in the rescue group compared to the intervention group (2.8 vs. 2.0; Table 1).

**Figure 2:**
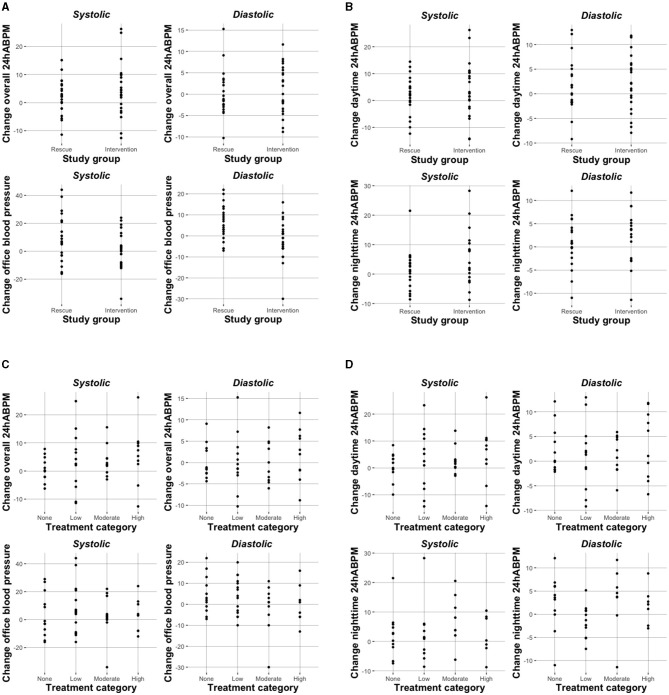
Change in blood pressure (mmHg) with respect to study group **(A,B)** and treatment category **(C,D)**.

Overall, we observed an increase in mean SBP and DBP from baseline to week 8 independent of study group (except for **Δ** office DBP intervention group), but this was statistically significant only for the intervention group for 24h-ABPM (**Δ**: 4.58, 95%CI: 0.30–8.86, mmHg) and systolic nighttime-ABPM (**Δ**: 5.72, 95%CI: 0.75, 10.70; mmHg), respectively (Table 2A). When looking at different treatment categories a similar trend toward increase in SBP and DBP was observed (Table 2B), with an exception for office DBP for moderate (**Δ**: −1.70, 95%CI: −10.02, 6.62; mmHg) and high treatment categories (**Δ**: −0.13, 95%CI: −7.86, 7.61; mmHg), and for diastolic nighttime-ABPM in the low sodium bicarbonate group (**Δ**: −1.39, 95%CI: −4.33, 1.54; mmHg). Individual change of blood pressure with respect to study group and treatment category are depicted in Figures 2A,B.

**Table 2B:** Mean office and 24h-ambulatory blood pressure measurements (± SD, mmHg) with respect to treatment categories and point in time and mean blood pressure change (95%CI, mmHg).

**Treatment category**		**Systolic blood pressure**			**Diastolic blood pressure**		
	***n***	**Baseline**	**Week 8**	**Change (95% CI)**	**Baseline**	**Week 8**	**Change (95% CI)**
**24h-ABPM**							
None	11	121 ± 12.1	122 ± 11	0.72 (−2.27 to 3.71)	75 ± 3.8	77 ± 5.9	1.71 (−1.38 to 4.81)
Low	15	124 ± 9.9	127 ± 12.5	3.03 (−2.27 to 8.34)	78 ± 7.8	79 ± 7.3	0.4 (−2.53 to 3.33)
Moderate	8	128 ± 8.7	134 ± 12.4	6.31 (−3.26 to 15.88)	80 ± 6.1	83 ± 7.4	3.7 (−0.53 to 8.01)
High	9	120 ± 11.1	124 ± 9.1	4.44 (0.17 to 8.71)	80 ± 7.8	81 ± 4.7	1.83 (−2.35 to 6.01)
			*p*-value	0.214		*p*-value	0.488
**Daytime-ABPM**							
None	11	123 ± 13.4	124 ± 9.7	0.72 (−2.81 to 4.24)	76 ± 3.6	79 ± 6.3	2.35 (−0.92 to 5.62)
Low	15	125 ± 10.4	128 ± 11.2	2.69 (−2.96 to 8.34)	79 ± 8.2	80 ± 7.9	0.96 (−2.48 to 4.4)
Moderate	8	130 ± 10.7	135 ± 13.2	4.78 (−5.21 to 14.78)	81 ± 7.7	84 ± 7.5	2.75 (−1.85 to 7.36)
High	9	121 ± 9.9	125 ± 8.1	4.67 (−0.23 to 9.57)	80 ± 7.5	83 ± 5.2	2.53 (−2.42 to 7.48)
			*p*-value	0.261		*p*-value	0.768
**Nighttime-ABPM**							
None	11	117 ± 11.4	119 ± 14.1	2.45 (−2.8 to 7.71)	70 ± 8	73 ± 7.7	2.53 (−1.55 to 6.61)
Low	14	121 ± 10.9	123 ± 15.8	1.96 (−3.18 to 7.10)	76 ± 8.3	75 ± 8.5	−1.39 (−4.33 to 1.54)
Moderate	6	124 ± 8.9	133 ± 12.6	8.18 (−2.77 to 19.13)	75 ± 7.3	80 ± 9.7	4.39 (−0.56 to 9.35)
High	6	112 ± 11.2	116 ± 11.2	3.95 (−1.87 to 9.76)	73 ± 8.7	76 ± 7.8	2.68 (−1.18 to 6.55)
			*p*-value	0.396		*p*-value	0.547
**Office BP**							
None	12	129 ± 14.9	131 ± 16.5	3.42 (−6.62 to 13.45)	78 ± 17.3	79 ± 14.9	4.58 (−1.18 to 10.35)
Low	15	125 ± 18.3	133 ± 13.5	8.07 (−1.68 to 17.82)	76 ± 11.2	81 ± 7.8	4.07 (−0.84 to 8.98)
Moderate	10	136 ± 9.4	139 ± 15.7	3.2 (−8.04 to 14.44)	82 ± 6.7	81 ± 14.5	−1.7 (−10.02 to 6.62)
High	8	131 ± 16.6	132 ± 21	3.38 (−6.97 to 13.72)	87 ± 13.7	84 ± 19.2	−0.13 (−7.86 to 7.61)
			*p*-value	0.831		*p*-value	0.128

*Missings: 24h-ABPM and daytime-ABPM 2 none, 2 moderate; Nighttime-ABPM: 2 none, 4 moderate, 2 high; Office BP 1 none, 1 high*.

*Change between baseline and week 8 for respective treatment category was tested by a paired t-test, difference in change across treatment categories was tested by an analysis of variances (ANOVA)*.

*Treatment category: none, no sodium bicarbonate: low (1–37.9 mg/kg/day), moderate (38–79.9 mg/kg/day), high (≥80 mg/kg/day)*.

*24h-ABPM, 24 h ambulatory blood pressure monitoring; Daytime ABPM, from 8:00 until 22:00 ambulatory blood pressure monitoring; Nighttime ABPM, from 22:01 until 7:59 ambulatory blood pressure monitoring; Office BP, office blood pressure measurement; 95% CI, 95% confidence interval*.

In all 43 randomized subjects with sufficient 24h-ABPM neither systolic 24h-ABPM (2.522; 95%CI: −2.364, 7.408; mmHg) nor diastolic 24h-ABPM (0.868; 95%CI: −2.411, 4.147; mmHg) was affected by study group allocation (Table 3A). Adjustment for change of estimated glomerular filtration rate and change of antihypertensive medication did only slightly change estimates. In contrast, office BP measurements showed a decrease in BP for subjects being randomized to the intervention group (SBP: −8.241, 95%CI: −17.808, 1.327; DBP: −8.94, 95%CI: −14.458, −3.421; mmHg), however, only statistically significant for DBP.

**Table 3A:** Association of systolic and diastolic blood pressure change (mmHg) with respect to study group.

		**Regression models**					
		**Crude**		**Adjusted I**		**Adjusted II**	
	***n***	**β**	**95% CI**	**β**	**95% CI**	**β**	**95% CI**
**Δ** **24h-ABPM**	43						
Systolic		2.522	−2.364 to 7.408	2.585	−2.361 to 7.532	2.632	−2.515 to 7.800
Diastolic		0.868	−2.411 to 4.147	0.856	−2.469 to 4.182	0.797	−2.658 to 4.252
**Δ** **Daytime-ABPM**	43						
Systolic		1.816	−3.411 to 7.042	1.905	−3.379 to 7.189	2.119	−3.362 to 7.599
Diastolic		0.976	−2.437 to 4.388	0.918	−2.532 to 4.368	0.925	−2.654 to 4.504
**Δ** **Nighttime-ABPM**	37						
Systolic		4.231	−1.069 to 9.531	4.146	−1.238 to 9.529	4.018	−1.496 to 9.531
Diastolic		1.470	−2.069 to 5.009	1.214	−2.230 to 4.659	1.303	−2191 to 4.797
**Δ** **Office BP**	45						
Systolic		−7.289	−16.203 to 1.626	−7.296	−16.344 to 1.751	−8.241	−17.808 to 1.327
Diastolic		−8.19	−13.375 to −3.004	−8.35	−13.577 to −3.124	−8.94	−14.458 to −3.421

*Crude, intervention group (target ${\text{HCO}}_{3}^{-}$ 24 ± 1 mmol/L) vs. rescue group [reference; group (target ${\text{HCO}}_{3}^{-}$ 20 ± 1 mmol/L)]*.

*Adjusted I, Crude + change of eGFR from baseline to week 8 (mL/min per 1.73 m^2^).*

*Adjusted II, Adjusted I + increase and decrease of antihypertensive medication vs. no change of antihypertensive medication*.

*24h-ABPM, 24 h ambulatory blood pressure monitoring; Daytime ABPM, from 8:00 until 22:00 ambulatory blood pressure monitoring; Nighttime ABPM, from 22:01 until 7:59 ambulatory blood pressure monitoring; Office BP, office blood pressure measurement; β, point estimate (mmHg); 95% CI, 95% confidence interval*.

When looking at the effect of individual sodium bicarbonate dose on 24h-ABPM, the fully adjusted model suggested an increase of 0.047 (95%CI: −0.026, 0.119) mmHg by each mg/kg *per* day increase of sodium bicarbonate dose. None of the estimates reached statistical significance, however, the opposite direction of effects for office BP vs. 24h-ABPM remained present (Table 3B).

**Table 3B:** Association of systolic and diastolic blood pressure change (mmHg) with respect to sodium bicarbonate dose (mg/kg body weight).

		**Regression models**					
		**Crude**		**Adjusted I**		**Adjusted II**	
	***n***	**β**	**95% CI**	**β**	**95% CI**	**β**	**95% CI**
**Δ** **24h-ABPM**	43						
Systolic		0.047	−0.022 to 0.116	0.047	−0.023 to 0.117	0.047	−0.026 to 0.119
Diastolic		0.021	−0.026 to 0.068	0.021	−0.026 to 0.068	0.023	−0.026 to 0.071
**Δ** **Daytime-ABPM**	43						
Systolic		0.046	−0.028 to 0.119	0.045	−0.03 to 0.12	0.046	−0.033 to 0.122
Diastolic		0.013	−0.036 to 0.062	0.013	−0.036 to 0.063	0.015	−0.036 to 0.066
**Δ** **Nighttime-ABPM**	37						
Systolic		0.044	−0.037 to 0.124	0.044	−0.038 to 0.125	0.049	−0.036 to 0.133
Diastolic		0.026	−0.027 to 0.079	0.026	−0.025 to 0.077	0.022	−0.030 to 0.075
**Δ** **Office BP**	45						
Systolic		−0.024	−0.161 to 0.112	−0.024	−0.163 to 0.114	−0.036	−0.183 to 0.11
Diastolic		−0.067	−0.15 to 0.016	−0.067	−0.151 to 0.017	−0.068	−0.157 to 0.022

*Crude, sodium bicarbonate dose (mg /kg body weight)*.

*Adjusted I, Crude + change of eGFR from baseline to week 8 (mL/min per 1.73 m^2^)*.

*Adjusted II, Adjusted I + increase and decrease of antihypertensive medication vs. no change of antihypertensive medication*.

*24h-ABPM, 24 h ambulatory blood pressure monitoring; Daytime ABPM, from 8:00 until 22:00 ambulatory blood pressure monitoring; Nighttime ABPM, from 22:01 until 7:59 ambulatory blood pressure monitoring; Office BP, office blood pressure measurement; β, point estimate (mmHg); 95% CI, 95 % confidence interval*.

When treatment categories low, moderate, and high were compared against no sodium bicarbonate treatment we couldn't show a clear dose-dependent effect on blood pressure and none of the models were statistically significant. Again, when looking at office BP a higher does of sodium bicarbonate was associated with a decrease in BP (n.s.; Table 3C and Figures 2C,D). In Supplementary Tables 1A,B we present age- and diuretic use- adjusted estimates for the relation of sodium bicarbonate loading and blood pressure.

**Table 3C:** Association of systolic and diastolic blood pressure change (mmHg) with respect to treatment category (low, moderate, high; reference, none).

**Treatment category**		**Crude**				**Adjusted I**				**Adjusted II**			
		**Systolic**		**Diastolic**		**Systolic**		**Diastolic**		**Systolic**		**Diastolic**	
	***n***	**β**	**95% CI**	**β**	**95% CI**	**β**	**95% CI**	**β**	**95% CI**	**β**	**95% CI**	**β**	**95% CI**
**24h-ABPM**													
Low	15	2.316	−4.095 to 8.726	−0.63	−4.953 to 3.693	2.21	−4.332 to 8.752	−0.601	−5.016 to 3.814	2.212	−4.515 to 8.939	−0.55	−5.08 to 3.98
Moderate	8	5.589	−1.914 to 13.092	1.852	−3.209 to 6.912	5.534	−2.074 to 13.142	1.867	−3.268 to 7.0	5.706	−2.331 to 13.744	1.908	−3.504 to 7.32
High	9	3.722	−3.536 to 10.98	0.948	−3.947 to 5.843	3.654	−3.712 to 11.02	0.966	−4.005 to 5.938	3.607	−4.016 to 11.231	1.108	−4.025 to 6.242
**Daytime-ABPM**													
Low	15	1.972	−4.919 to 8.862	−1.387	−5.915 to 3.141	1.785	−5.238 to 8.807	−1.246	−5.858 to 3.366	1.764	−5.437 to 8.965	−1.177	−5.902 to 3.548
Moderate	8	4.064	−4.001 to 12.13	0.404	−4.896 to 5.705	3.966	−4.201 to 12.132	0.479	−4.885 to 5.842	4.351	−4.254 to 12.955	0.617	−5.029 to 6.262
High	9	3.951	−3.851 to 11.753	0.177	−4.95 to 5.304	3.832	−4.076 to 11.739	0.267	−4.925 to 5.46	3.652	−4.509 to 11.813	0.433	−4.922 to 5.787
**Nighttime-ABPM**													
Low	14	−0.495	−7.143 to 6.150	−3.920	−8.003 to 0.162	−0.336	−7.110 to 6.438	−3.540	−7.538 to 0.458	−0.262	−7.153 to 6.628	−3.579	−7.648 to 0.491
Moderate	6	5.728	−2.645 to 14.102	1.860	−3.28 to 7.003	5.748	−2.733 to 14.229	1.908	−3.073 to 6.914	6.083	−2.672 to 14.839	1.739	−3.431 to 6.910
High	6	1.492	−6.881 to 9.866	0.150	−4.992 to 5.293	1.588	−6.050 to 10.080	0.378	−4.638 to 5.390	2.255	−6.482 to 10.981	−0.112	−5.171 to 5.148
**Office BP**													
Low	15	4.65	−7.425 to 16.725	−0.517	−7.847 to 6.813	4.645	−7.653 to 16.942	−0.311	−7.752 to 7.131	4.507	−8.053 to 17.067	−0.29	−7.906 to 7.327
Moderate	10	−0.217	−13.567 to 13.133	−6.283	−14.387 to 1.82	−0.219	−13.744 to 13.307	−6.205	−14.389 to 1.979	−1.139	15.431 to 13.153	−6.567	−15.233 to 2.1
High	8	−0.042	−14.273 to 14.189	−4.708	−13.347 to 3.93	−0.044	14.467 to 14.379	−4.606	−13.333 to 4.122	−0.96	−15.955 to 14.035	−4.616	−13.71 to 4.477

*Crude, treatment category of sodium bicarbonate: low (1–37.9 mg/kg/day), moderate (38–79.9 mg/kg/day), and high (≥80 mg/kg/day) vs. no treatment (reference); Adjusted I, Crude + change of eGFR from baseline to week 8 (mL/min per 1.73 m^2^); Adjusted II, Adjusted I + increase and decrease of antihypertensive medication vs. no change of antihypertensive medication*.

*24h-ABPM, 24 h ambulatory blood pressure monitoring; Daytime ABPM, from 8:00 until 22:00 ambulatory blood pressure monitoring; Nighttime ABPM, from 22:01 until 7:59 ambulatory blood pressure monitoring; Office BP, office blood pressure measurement; β, point estimate (mmHg); 95% CI, 95 % confidence interval*.

### Adverse Events, Antihypertensive Medication, and Compliance

Overall mean body weight changed by −0.2 (±3.64) kg, 0.13 (±3.52) kg in the intervention group and −0.5 (±3.8) kg in the rescue group (*p* = 0.717) from baseline to week 8. In neither the intervention group nor the rescue group development or worsening of peripheral edema was observed.

In six (13%) patients antihypertensive medication changed during the 8 weeks of follow-up: In one subject a thiazide diuretic was started instead of calcium antagonist treatment, and in another a calcium antagonist was started (both 50% of max. daily dose; both intervention group). Angiotensin- converting-enzyme inhibitor (ACEi) dose was increased by 6.25% of max. daily dose, while furosemide was decreased and doxazosin was stopped in another subject in the rescue group. In the remaining three patients (one intervention group) with treatment change doses were decreased (furosemide, ACEi and thiazide, ACEi and calcium antagonist). Overall, use of classes of antihypertensives decreased to 1.9 *per* person in the intervention group and 2.5 *pe*r person in the rescue group from baseline until week 8.

Study medication was well-tolerated and compliance was good, except for one female who didn't want to take as many capsules as would have been necessary to reach the target level (nine *per* day). Serum bicarbonate on average increased in the intervention group by 3.9 (95%CI: 3.04, 4.71) mmol/L and by 1.5 (95%CI: 0.47, 2.56) mmol/L in the rescue group. At week 8 median urinary pH was 7 (IQR: 6.5–7.25) in the intervention and 5 (IQR: 5–5.6) in the rescue group. Urine sodium excretion was only determined at week 4, but increased on average by 32.77 (95%CI: −4.44, 69.99) mmol/24 h in the intervention group and by 12.47 (95%CI: −7.24, 32.18) mmol/24 h in the rescue group.

## Discussion

To the best of our knowledge this is the first study investigating the effect of sodium bicarbonate treatment on arterial blood pressure using the gold standard measurement technique of 24h-ABPM. Overall, we did not observe a statistically significant effect on blood pressure by sodium bicarbonate treatment. Change in antihypertensive medication and weight gain was negligible. However, 24h-ABPM increased consistently by approximately two to five mmHg between baseline and week 8 (Table 2A). This increase was present in systolic and diastolic measurements and was somewhat more distinct in the intervention group compared to the rescue group, as indicated by the positive regression coefficients (Table 3A). Regardless, we could not establish a dose-dependent effect of sodium bicarbonate on blood pressure, as the magnitude of BP increase didn't consistently rise by increasing dose category (Table 2B). For systolic 24h-ABPM effect sizes (β) approximately suggest doubling of BP increase from low to a moderate sodium bicarbonate dose in relation to subjects receiving no sodium bicarbonate, but decreased in the high bicarbonate dose group (Table 3C). Notably, none of our results reached statistical significance and the observed trends might be due to random error.

Striking are the differences between office and ambulatory measurements. Mean baseline office SBP was relatively low in the rescue group and did increase to comparable levels until week 8 (Figure 2A). This led to negative effect sizes for regression models looking at office BP change, suggesting a decrease of BP with increasing sodium bicarbonate dose. Our results highlight the limitations of office BP measurements even though when performed by a well-trained nurse using a standardized protocol.

The amount of sodium administered while correcting metabolic acidosis with sodium bicarbonate is substantial. In the current study the intervention group received a median daily dose of 63 mg/kg *per* day (0.76 mEq/kg *per* day), which translates to 6 capsules à 840 mg *per* day for an individual weighing 80 kg. In light of the recent evidence showing improvement of renal function and more importantly survival in sodium bicarbonate supplemented CKD patients with chronic acidosis (1), the uncertainty of safety with regard to worsening of arterial hypertension and changes in fluid status is prevailing in clinical practice (5).

Looking at our regression model a daily dose of 63 mg/kg sodium bicarbonate would lead to an on average increase of 3 mmHg systolic and 1.3 mmHg diastolic 24h-ABPM [systolic 24h-ABPM: +4.7 (95%CI: −2.2 to +11.6)] mmHg by 100 mg/kg *per* day orally administered sodium bicarbonate (Table 3B).

The question whether sodium or chloride is the driving force for arterial hypertension in salt-sensitive individuals is not novel. Indisputable is the recommendation for a low sodium chloride diet of <5 g *per* day [corresponding to <2 g (<90 mmol) of sodium *per* day] in patients with arterial hypertension and CKD (11). The average effect on SBP and DBP for the suggested limitation of salt intake is 4.7 mmHg (95%CI: 2.2–7.2) and 2.5 mmHg (95%CI: 1.8–3.3), respectively (12). The relationship between elevated ambulatory BP and all-cause and cardiovascular mortality is well-established (13). Although our estimates were not statistically significant, the shown blood pressure-reducing effect of a salt-restricted diet would be almost outweighed by the blood pressure-increasing effect of a regularly necessary sodium bicarbonate dose to counteract acidosis in CKD. However, a set of small randomized controlled trials including 5–10 subjects each specifically studied the effect of NaCl on blood pressure, sodium retention, and weight gain compared to NaHCO_3_ and sodium citrate, respectively (14–18). Under these experimental conditions, which included a very-low dietary sodium load of 10 mEq *per* day a sodium load accompanied by bicarbonate or citrate led to weight increase and sodium retention but not to increase of blood pressure. These observations are supported by animal experiments suggesting that the BP increasing effect is sufficiently induced by chloride alone (19–21), but more pronounced for NaCl (22, 23). The renin-angiotensin system, which is usually suppressed after a NaCl load and rise in blood pressure, is not affected by non-chloride sodium salts, both in animals (24–26) and men (27). However, with more realistic dietary salt loading of either 200 mEq *per* day of NaCl or 100 mEq *per* day of NaCl plus 100 mEq *per* day of NaHCO_3_ blood pressure increase was comparable between groups in a randomized crossover trial in 6 patients with CKD stage G5 (28). Furthermore, more recent studies tested the effect of sodium bicarbonate on blood pressure as a secondary outcome, but used placebo as a control substance. de Brito-Ashurst et al. observed no difference in blood pressure, but increased antihypertensives use in the study group (61 vs. 48%) after 24 months (2). Also no difference in BP was seen in a study testing sodium citrate compared to controls after over 30 months (29). A recent study in CKD stage G3 and G4 patients with a bicarbonate lower than 22 at baseline and a target bicarbonate of 24 to 26 for the intervention group, recorded “worsening of hypertension” in 35.1 % of the intervention group vs. 21% in the control group [*p* = 0.13; (30)]. “Worsening of edema” and diuretic use was significantly more frequent with a mean dose of sodium bicarbonate of 2.3 g/day (0.5 mEq/kg body weight *per* day) in the intervention group after the 6 months follow-up. Jeong et al. followed CKD stage G4 and G5 subjects over 10 months (31). The mean dose of sodium bicarbonate was 0.58 ± 0.42 mEq/kg in the intervention group. Blood pressure did neither change in CKD stage G4 nor stage G5 (treatment group: 3.4 ± 16.3/0.7 ± 11.5 mmHg; controls: −2.1 ± 22.3/−1.8 ± 12.3 mmHg) with similar use of antihypertensives and loop diuretics across groups. However, in both studies a trend toward worsening of arterial hypertension was seen and observed effects are comparable with our results in magnitude. Of note, daily average sodium bicarbonate load in our study was much higher in the intervention group, which could indicate that the increase in BP is due to other reasons independent of sodium bicarbonate load. The so far largest trial investigating sodium bicarbonate treatment in CKD, the UBI study, presented little information on BP monitoring and change during the course of the 36 months follow-up, but did not observe worsening of hypertension (1). On the contrary, the recently published BASE Pilot trial (32) observed a significant increase in SBP after 12 weeks of high-dose sodium bicarbonate treatment compared to the low-dose and placebo group. Although not statistically significant this effect remained until week 32, when mean SBP was lower in the high-dose than the low-dose group but still higher than the placebo group. Altogether this might give a hint toward a dose-dependent effect. Like in our study, Abramowitz et al. observed a non-dose dependent effect on SBP and DPB in their dose-finding study. Sodium bicarbonate was increased every 2 weeks by ~0.3 mEq *per* kg ideal body weight (33).

In light of the possible blood pressure increasing effect of sodium bicarbonate, the set of studies by Goraya et al. (34–37) who used a fruit and vegetable-rich diet to counteract acidosis in CKD, is even more remarkable. In patients with hypertensive nephropathy and different stages of CKD SBP was lower in subjects treated with the dietary intervention compared to sodium bicarbonate or standard care. Further indicators of cardiovascular risk as well-improved in the dietary group (34). However, whether sodium bicarbonate supplementation ameliorates cardiovascular outcomes ought to be shown (38, 39).

Taken together, our study is in line with former reports showing a statistically non-significant trend toward an increase of blood pressure in sodium bicarbonate treated patients. However, like others (32, 33) we did not observe a dose-dependent effect as is shown in Figure 2. Ultimately, we don't know the reason behind the slight increase in BP in our cohort. One can speculate that individual dietary sodium chloride consumption might have mediated increase in blood pressure in some individuals.

Our study was limited by a relatively small sample size, in addition to four insufficient daytime- and 10 nighttime-ABPMs. Furthermore, we did not perform dietary assessment in particular salt intake, but our study subjects received standard dietary assistance by a trained dietitian as needed. Correction of acidosis by sodium bicarbonates results in high pill burden, suggesting incomplete compliance. However, the short duration of follow-up gives confidence that studied subjects followed prescriptions. Objective measures such as urine pH, ${\text{HCO}}_{3}^{-}$ change and urinary sodium excretion suggested sufficient compliance.

In conclusion, sodium loading accompanied by sodium bicarbonate supplementation to counterbalance chronic metabolic acidosis in CKD did not significantly increase BP. Nevertheless, BP of sodium bicarbonate treated CKD patients should be monitored and 24h-ABPM might be recommended for this vulnerable patient cohort.

## Data Availability Statement

The raw data supporting the conclusions of this article will be made available by the authors, without undue reservation.

## Ethics Statement

The studies involving human participants were reviewed and approved by Ethics Committee of the Medical University of Vienna. The patients/participants provided their written informed consent to participate in this study.

## Author Contributions

MG, SR, and GS-P designed the study. CA, SR, AR, CS, MF, MG, and GS-P conducted the study and collected the data. MG, CA, DC, and GS-P drafted the manuscript. All authors contributed to the article and approved the submitted version.

## Conflict of Interest

The authors declare that the research was conducted in the absence of any commercial or financial relationships that could be construed as a potential conflict of interest.

## Publisher's Note

All claims expressed in this article are solely those of the authors and do not necessarily represent those of their affiliated organizations, or those of the publisher, the editors and the reviewers. Any product that may be evaluated in this article, or claim that may be made by its manufacturer, is not guaranteed or endorsed by the publisher.
