# Supplementary material for: Effect of Oral Sodium Bicarbonate Treatment on 24-Hour Ambulatory Blood Pressure Measurements in Patients With Chronic Kidney Disease and Metabolic Acidosis
Source: Front Med (Lausanne). 2021 Sep 6;8:711034. doi: 10.3389/fmed.2021.711034 (PMC8450526; doi:10.3389/fmed.2021.711034)
Supplement: Supplementary file 1 [file Table_1.pdf]

Supplemental table 1. (A) Association of systolic and diastolic blood pressure change (mmHg) with respect to study group.

		Regression models					
		**Crude**		**Adjusted Ib**		**Adjusted IIb**	
	n	β	95% CI	β	95% CI	β	95% CI
**Δ 24h-ABPM**	43						
Systolic		2.522	-2.364 to 7.408	2.497	-2.488 to 7.482	1.769	-3.414 to 6.952
Diastolic		0.868	-2.411 to 4.147	0.804	-2.533 to 4.16	1.04	-2.485 to 4.565
**Δ Daytime-ABPM**	43						
Systolic		1.816	-3.411 to 7.042	1.808	-3.515 to 7.132	1.25	-4.32 to 6.821
Diastolic		0.976	-2.437 to 4.388	0.856	-2.621 to 4.332	0.949	-2.711 to 4.61
**Δ Nighttime-ABPM**	37						
Systolic		4.231	-1.069 to 9.531	4.077	-1.416 to 9.57	3.895	-1.807 to 9.598
Diastolic		1.470	-2.069 to 5.009	1.467	-1.959 to 4.894	1.824	-1.691 to 5.339
**Δ Office BP**	45						
Systolic		-7.289	-16.203 to 1.626	-7.447	-16.655 to 1.761	-8.141	-17.833 to 1.327
Diastolic		-8.19	-13.375 to -3.004	-8.355	-13.679 to -3.031	-8.97	-14.308 to -3.091

Crude= intervention group (target HCO_3^- 24±1 mmol/L) vs. rescue group (reference; group (target HCO_3^- 20±1 mmol/L);
Adjusted Ib = Crude + change of eGFR from baseline to week 8 (mL/min *per* 1.73m^2) + age (years)
Adjusted IIb= Adjusted I + use of diuretics.
24h-ABPM, 24 hours ambulatory blood pressure monitoring; Daytime ABPM, from 8:00 until 22:00 ambulatory blood pressure monitoring; Nighttime ABPM, from 22:01 until 7:59 ambulatory blood pressure monitoring; Office BP, office blood pressure measurement; β = point estimate (mmHg); 95% CI, 95 % confidence interval.

Supplemental table 1. (B) Association of systolic and diastolic blood pressure change (mmHg) with respect to sodium bicarbonate dose (mg/kg body weight).

		Regression models					
		**Crude**		**Adjusted Ib**		**Adjusted IIb**	
	n	β	95% CI	β	95% CI	β	95% CI
**Δ 24h-ABPM**	43						
Systolic		0.047	-0.022 to 0.116	0.049	-0.022 to 0.119	0.041	-0.031 to 0.113
Diastolic		0.021	-0.026 to 0.068	0.022	-0.025 to 0.07	0.025	-0.024 to 0.073
**Δ Daytime-ABPM**	43						
Systolic		0.046	-0.028 to 0.119	0.047	-0.03 to 0.122	0.042	-0.035 to 0.119
Diastolic		0.013	-0.036 to 0.062	0.014	-0.035 to 0.064	0.015	-0.036 to 0.067
**Δ Nighttime-ABPM**	37						
Systolic		0.044	-0.037 to 0.124	0.044	-0.038 to 0.127	0.042	-0.042 to 0.126
Diastolic		0.026	-0.027 to 0.079	0.025	-0.025 to 0.076	0.028	-0.023 to 0.079
Δ Office BP	45						
Systolic		-0.024	-0.161 to 0.112	-0.024	-0.164 to 0.115	-0.026	-0.171 to 0.119
Diastolic		-0.067	-0.15 to 0.016	-0.067	-0.152 to 0.018	-0.066	-0.157 to 0.022

Crude= sodium bicarbonate dose (mg /kg body weight);
Adjusted Ib = Crude + change of eGFR from baseline to week 8 (mL/min *per* 1.73m^2) + age (years);
Adjusted IIb= Adjusted I + use of diuretics.
24h-ABPM, 24 hours ambulatory blood pressure monitoring; Daytime ABPM, from 8:00 until 22:00 ambulatory blood pressure monitoring; Nighttime ABPM, from 22:01 until 7:59 ambulatory blood pressure monitoring; Office BP, office blood pressure measurement; β = point estimate (mmHg); 95% CI, 95 % confidence interval
